# The *cagE* gene sequence as a diagnostic marker to identify JP2 and non-JP2 highly leukotoxic *Aggregatibacter actinomycetemcomitans* serotype b strains

**DOI:** 10.1080/20002297.2017.1325257

**Published:** 2017-06-09

**Authors:** Anders Johansson, Rolf Claesson, Carola Höglund Åberg, Dorte Haubek, Jan Oscarsson

**Affiliations:** ^a^ Divisions of Oral Microbiology, Umeå University, Umeå, Sweden; ^b^ Molecular Periodontology, Department of Odontology, Faculty of Medicine, Umeå University, Umeå, Sweden; ^c^ Section for Pediatric Dentistry, Department of Dentistry, Oral Health Aarhus University, Aarhus, Denmark

## Abstract

*Aggregatibacter actinomycetemcomitans* is associated with aggressive forms of periodontitis, and with systemic infections such as endocarditis. The *cagE* gene encodes a ≈39-kDa putative exotoxin expressed by *A. actinomycetemcomitans*. The prevalence of *cagE*, and its significance in periodontal disease, has not yet been thoroughly evaluated. In the present study, the role of the *cagE* gene as a diagnostic marker has been investigated.

We have used PCR, and whole genome sequencing data to determine the prevalence of *cagE* in *A. actinomycetemcomitans* based on analysis of (i) 249 isolates, collected and cultivated in a Ghanaian longitudinal cohort study, (ii) a serotype b collection of 19 strains, and (iii) the 36 *A. actinomycetemcomitans* genomes available in the NCBI database.

Whereas *cagE* was absent in the other serotypes, this gene sequence was linked to a virulent and highly leukotoxic group of serotype b strains, including both JP2 and non-JP2 genotypes.

We therefore propose that *cagE* has a potential to be used as a PCR-based gene marker for the identification of a virulent and highly leukotoxic group of serotype b strains, including both JP2 and non-JP2 genotypes. This might be of importance in the risk assessment of the development of periodontal attachment loss in young individuals.

